# Seizures elicited by transcorneal 6 Hz stimulation in developing rats

**DOI:** 10.1371/journal.pone.0313681

**Published:** 2025-01-03

**Authors:** Pavel Mareš, Cameron S. Metcalf, Jiří Baloun, Hana Kubová

**Affiliations:** 1 Department of Developmental Epileptology, Institute of Physiology, Czech Academy of Sciences, Prague, Czech Republic; 2 Department of Pharmacology and Toxicology, College of Pharmacy, University of Utah, Salt Lake City, UT, United States of America; 3 Institute of Rheumatology, Prague, Czech Republic; University of Modena and Reggio Emilia, ITALY

## Abstract

Seizures elicited by corneal 6-Hz stimulation are widely acknowledged as a model of temporal lobe seizures. Despite the intensive research in rodents, no studies hint at this model in developing animals. We focused on seven age groups of both male and female rats. Biphasic pulses with 0.3 ms duration and current intensities from 20 to 80 mA were applied transcorneally for 3 s to calculate threshold intensities for individual age groups. Threshold stimulation intensity necessary for elicitation of clonic seizures was highly age- and sex-dependent. The highest threshold was observed in the youngest (15-day-old) group then it decreased to the age of 25 days and increased again up to adulthood. The threshold current tended to be lower in females of all age groups. The incidence of convulsive seizures increased with stimulation intensity up to postnatal day 25 in either sex. In rats of 31 days old and older convulsions occurred irregularly regardless of the stimulation current and sex. For subsequent analysis, the animals were categorized into two groups: juveniles, aged 15 to 25 days, and adolescents/adults, aged 31 days and older. Our statistical analyses revealed an increased risk of convulsions after the stimulation with higher intensities in juvenile but not adolescent/adult rats. Females tended to be more sensitive to the stimulation with lower currents than males. Seizure severity was higher in females 18- to 25-day old compared to males of the same age and the seizure duration increased with stimulation intensities in juvenile but not adolescent/adult animals. The data extend the use of the rat 6 Hz model to immature animals and may be useful as a model of pediatric temporal lobe seizures.

## 1. Introduction

The 6 Hz model of temporal lobe epileptic seizures was described by Toman [1] in mice. Although it was abandoned because of the inconsistency of pharmacological sensitivity with clinically successful antiepileptic drugs [2], the revival of this model came after 50 years. Barton et al. [3] demonstrated its sensitivity to AEDs and their study started interest in this model. Subsequently, most studies were performed in mice [4] and the 6-Hz test in mice was added to a spectrum of tests used in the NIH Epilepsy Therapy Screening Program [5].

There are three intensities of stimulation used today in mice. Lower intensities (usually 22 and 32 mA) served for standard testing of new anticonvulsants. The highest intensity (44 mA) is taken for a model of therapy-resistant seizures [6]. Repeated transcorneal stimulation, resulting in kindling [7–9], was used for testing of antiepileptic drugs [4,10,11] as well as of the ketogenic diet [12].

Data for rats are scanty. Among a few experiments, only two important studies were performed in adult animals published in 2017 [13,14]. Esnault with collaborators [14] were focused on comparison of results in rats and mice using stimulation intensities from 24 to 44 mA. They demonstrated an increase in the proportion of seizing mice with increasing current intensity whereas the severity of seizures changed only moderately. In contrast, rat studies did show marked increases in both parameters with increasing stimulation current. Metcalf et al. [13] also presented a comparison of 35-day-old Sprague-Dawley rats with mice in the 6 Hz model and an extensive systematic pharmacological study. The efficacy of antiepileptic drugs in the 6 Hz model was compared with results in the maximal electroshock seizure test. These outcomes indicated a starting point for our study motivated by the fact that in contrast to many epilepsies starting at an early age [15] developmental data for the 6 Hz model are missing. It is in contrast with two models of limbic epileptic seizures–systemic administration of kainite [16–19] and intrahippocampal injection of kainite [20,21]. All these studies demonstrated a possibility of eliciting limbic seizures since the early stages of postnatal development but the variability of latency after systemic kainate and technically complicated intrahippocampal injections into immature brains decreased the utility of these two models for routine anticonvulsant testing.

Firstly, the analysis of the 6-Hz model required a screening of possible age specificities of the model and its adequacy for the testing of drugs for pediatric epilepsies and the comparison of male and female animals. The first data from seven age groups of developing male rats were presented at the American Epilepsy Society Meeting in Baltimore in December 2019 [22].

## 2. Material and methods

### 2.1. Animals

Experiments were performed in seven age groups of male and seven groups of freely cycling female Wistar rats (15, 18, 21, 25, 31, 45 and 60 days old). Animals younger than 15 days were not tested because they still have closed eyelids and surgical opening may cause significant stress. A total of 140 rats (70 male and 70 female animals) were used in this experiment, the animals were used only once for the series of seven stimulations. Individual age groups consisted of 10 rats. The animals were used only once for the series of seven stimulations. After finishing the experiment, the rats were sacrificed by an overdose of general aneethetic. The procedures involving animals and their care were conducted according to the ARRIVE guidelines https://www.nc3rs.org.uk/arrive-guidelines in compliance with national (Act No 246/1992 Coll.) and international laws and policies (EU Directive 2010/63/EU for animal experiments) and the National Institutes of Health guide for the care and use of Laboratory animals (NIH Publications No. 8023, revised 1978). Experiments were approved by the Animal Care and Use Committee of the Institute of Physiology of the Czech Academy of Sciences (No. 73–2016) to agree with all aforementioned directions and Czech Animal Protection Law.

### 2.2. Stimulation

Transcorneal stimulation was performed using silver electrodes on a plastic holder with a changeable distance between the electrodes. A Ugo Basile pulse generator ECT Unit 57800 was used in a constant current mode, and 6-Hz stimulation series with 0.3-ms square wave biphasic pulses were applied for 3 s. The intensity was increased in 10-mA steps from 20 to 80 mA, i.e. each animal was stimulated seven times. The interval between subsequent stimulations was 20 min. During this interval, the animals were in Plexiglas cages with wooden shavings, and cages with rats younger than 25 days were on a pad electrically heated to 35°C. Ten to five min before each stimulation a small drop of local anesthetic mesocaine (0.5%) was instilled into either eye = each animal received 14 drops during the more than 2-hour lasting experiment.

### 2.3. Evaluation

The animals were observed during stimulation, seizures and at least 30 seconds thereafter. If seizures did not appear observations continued for 30 seconds following stimulation. Seizure duration, pattern and intensity were registered. For quantification of the severity of seizures, Racine scale [23] was used. The lowest intensity eliciting seizures was taken as a threshold, and mean thresholds were calculated for each age group.

### 2.4. Statistics

The sample size was determined in advance based on previous experience and following the principles of the three Rs (Replacement, Reduction and Refinement; https://www.nc3rs.org.uk/the-3rs). All efforts were made to minimize the number of animals used and their suffering.

Outcome measures were prospectively selected. Data acquisition and analysis were performed by a blinded expeerimenter.

Data were analyzed using GraphPad Prism 9 (GraphPad Software, United States) software. Using the D’Agostino-Pearson normality test, all data sets were first analyzed to determine whether the values were derived from a Gaussian distribution. We used Type I ANOVA to identify the main effect of age and sex on threshold stimulation intensity. In this study, unless otherwise specified, the abbreviation ANOVA refers to Type I ANOVA for the analysis of variance. Whenever a significant association was identified, the data were subjected to Tukey’s post-hoc test.

Average duration measured from the end of stimulation and severity of seizures (plus standard deviation and standard error of the mean) were calculated for individual stimulation intensities in all age groups. Median and 95% confidence limits were also calculated.

To analyse longitudinal data, we employed generalized linear mixed-effects modelling (GLMM) for logistic regression and subjects as the random effect. Data were analyzed in R with RStudio and required packages [24–30]. Since we discovered high variability in the score among individual time points, we divided animals into two developmental categories: juvenile (P15-P25) and adolescence/adult (P31-P60), which improved the final model (e.g., R^2^). We fitted this model, using the convulsion occurrence as the outcome variable, and included stimulation threshold, sex, and developmental category as fixed effects. We also fitted a random-effects structure, which includes a random intercept for subjects (animal ID). Our model included the interaction effect of stimulation with gender or developmental category. To test the effect of explanatory variables on the occurrence of convulsion, the following model was used:

$$\begin{array}{ll} {convulsions}_{i} & ∼Binomial(n=1,{prob}_{convulsions=1}=\hat{P})\\ \;log\left[\frac{\hat{P}}{1-\hat{P}}\right] & =\alpha_{j[i]}+\beta_{1}(stimulation)+\beta_{2}({stage}_{adolescent/adult})+\beta_{3}({stage}_{adolescent/adult}\times stimulation)\\ \;\alpha_{j} & ∼N(\gamma_{0}^{\alpha }+\gamma_{1}^{\alpha }({sex}_{male})+\gamma_{2}^{\alpha }({sex}_{male}\times stimulation),\sigma_{\alpha_{j}}^{2}),\mathrm{for}\;\mathrm{animal}\;j=1,\ldots ,J \end{array}$$

where i—the presence of the convulsion; j–animal ID; β –regression coefficient; γ –random parameter. The effect of the explanatory variables was represented as the log odds ratio (logOR), which shows the strength and direction of the relationship between the variables and the probability of the outcome occurring. To evaluate the effect of the variables on the convulsion occurrence, we employed Type II ANOVA to estimate the effect of the variables. Afterwards, we performed pairwise comparisons, which were focused on specific comparisons between juveniles and adolescent/adult or males and females at each stimulation point. The significance level was considered p-value less than 0.05 without applying multiple testing corrections.

All statistical data are presented in (S1–S4 Tables).

## 3. Results

### 3.1. Behavior during stimulation

Clonic movements of forelimbs (Racine`s stage 3) were elicited during the 3 s stimulation period by all stimulation intensities in all male as well as female rats across ages. Their intensity increased with increasing stimulation current from minute clonic movements to marked movements of both forelimbs. If seizures were elicited, they started immediately after the end of stimulation.

### 3.2. Threshold stimulation intensity for induction of convulsive seizures (S5 Table)

**Fig 1:**
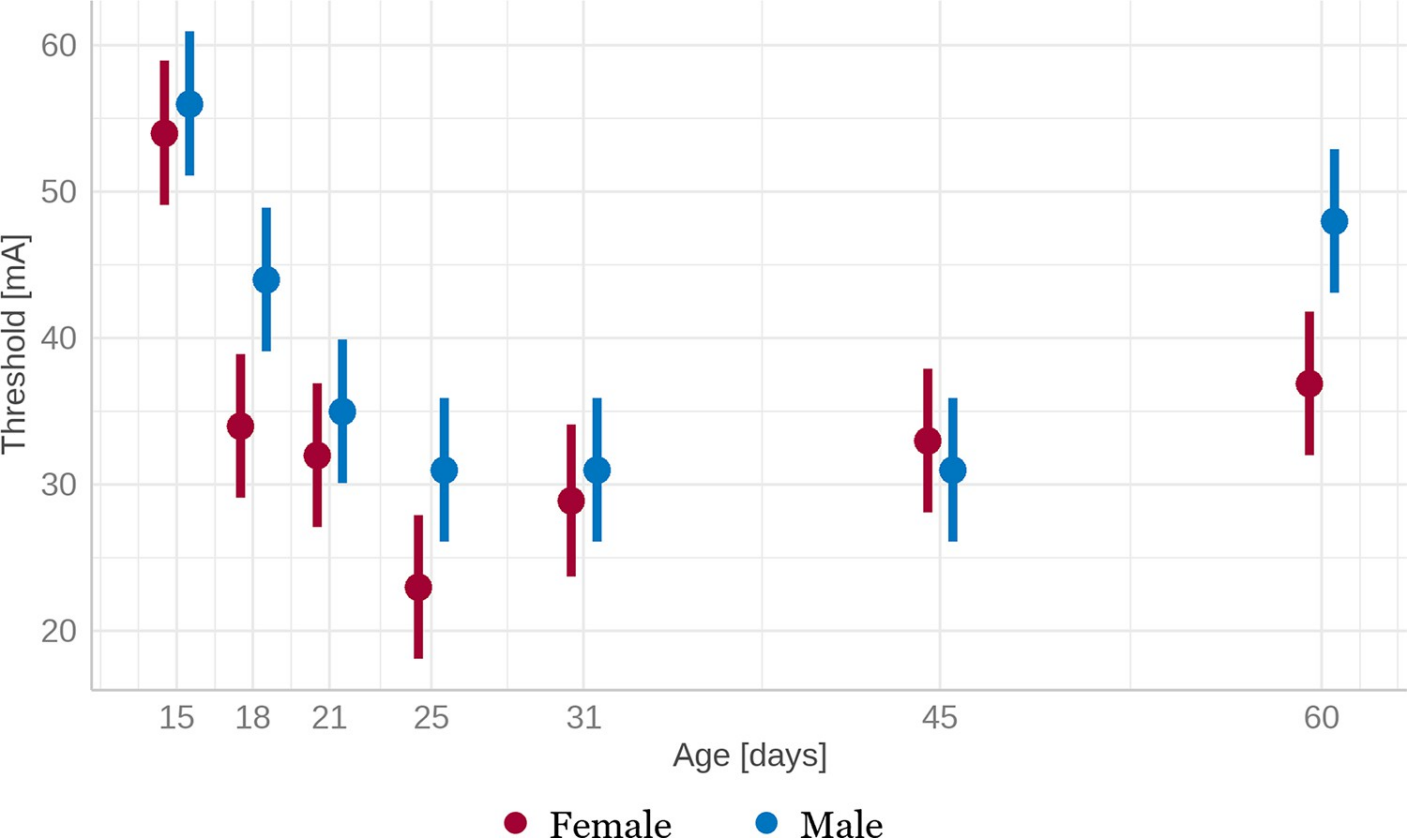
Age- and sex-related differences in the threshold stimulation intensity necessary to induce motor seizures (score 3–5). Axis x–age in days, axis y–threshold stimulation intensity in mA. Males in blue, Females in red. The error bars represent the 95% confidential intervals with the estimated means as a filled circle; both parameters were computed from the model.

Developmental pattern of threshold stimulation intensities necessary to elicit convulsive seizure exhibited a reversed J shape in rats of both sexes. ANOVA revealed a significant association of the threshold stimulation intensity with both age and sex (both p<0.001) but not on their interaction (p = 0.076). Post hoc analysis showed a significant difference between males and females in P18 and P60 animals. Regardless of sex, threshold stimulation intensities tended to be the highest in the youngest (P15) animals compared to other age groups. Also, females of all ages except P45 rats tended to be more sensitive to stimulation than males (Fig 1).

### 3.3. Incidence of convulsive seizures Score 3–5 (S6 and S7 Tables)

**Fig 2:**
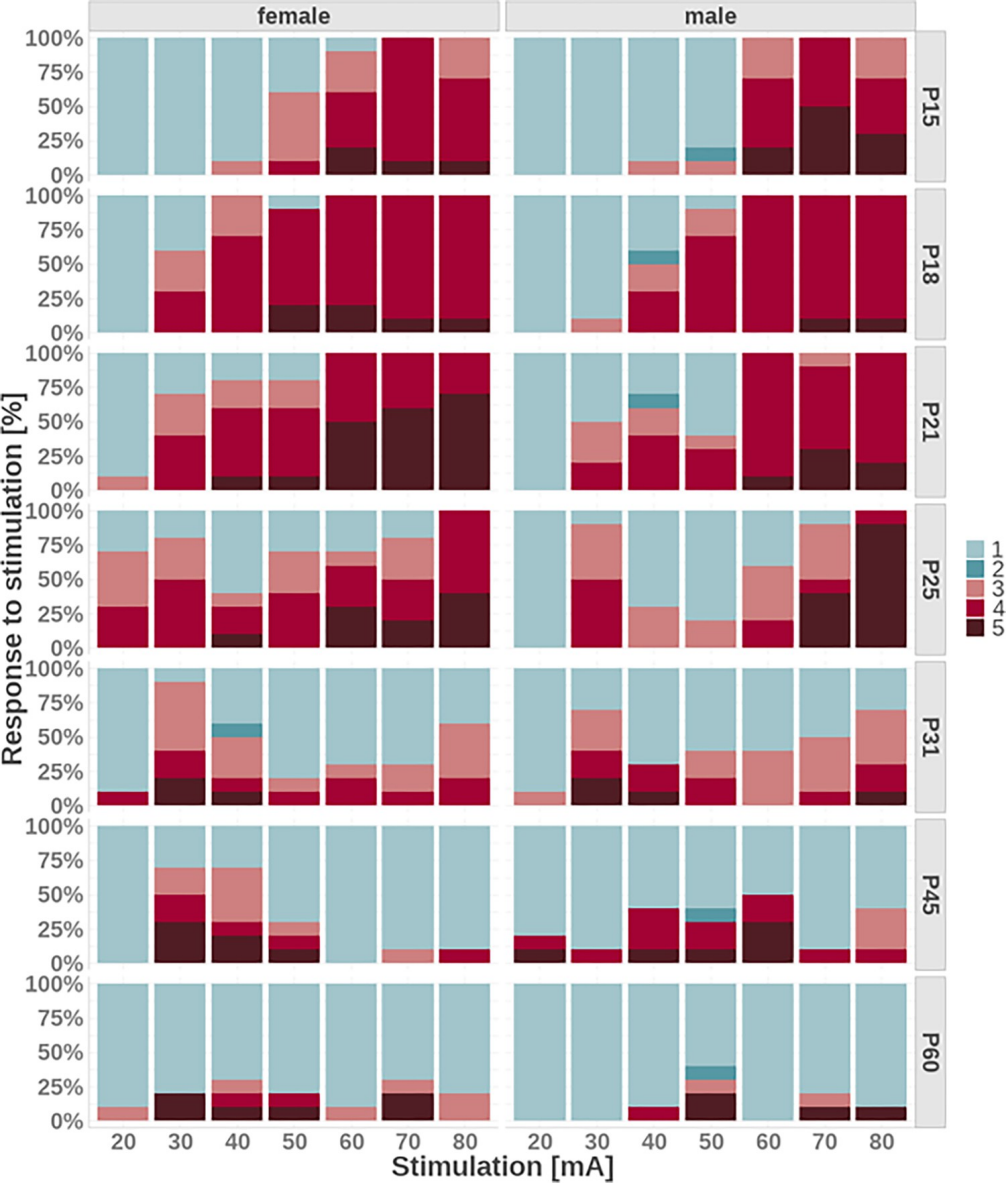
Seizure severity expressed as a score of 1–5. The plot shows proportions of the score values 1–5 (seizure severity) in percentage at each stimulation intensity and age for females or males. The shades of red indicate the severity of convulsions score (3–5). A lower score in blue indicates either no convulsions following the stimulation (1) or outlined motor seizures (i.e. with hyperlocomotion, score 2).

Juvenile, P15-P21 animals of both sexes exhibited progressively increased incidence of convulsive seizures with increasing stimulation intensity. P25 rats revealed a similar association only with stimulation intensities 50 mA and higher. The adolescent/adult animals (P31-P60) had an irregular incidence of convulsive seizures regardless of stimulation intensity and sex (Fig 2).

**Fig 3:**
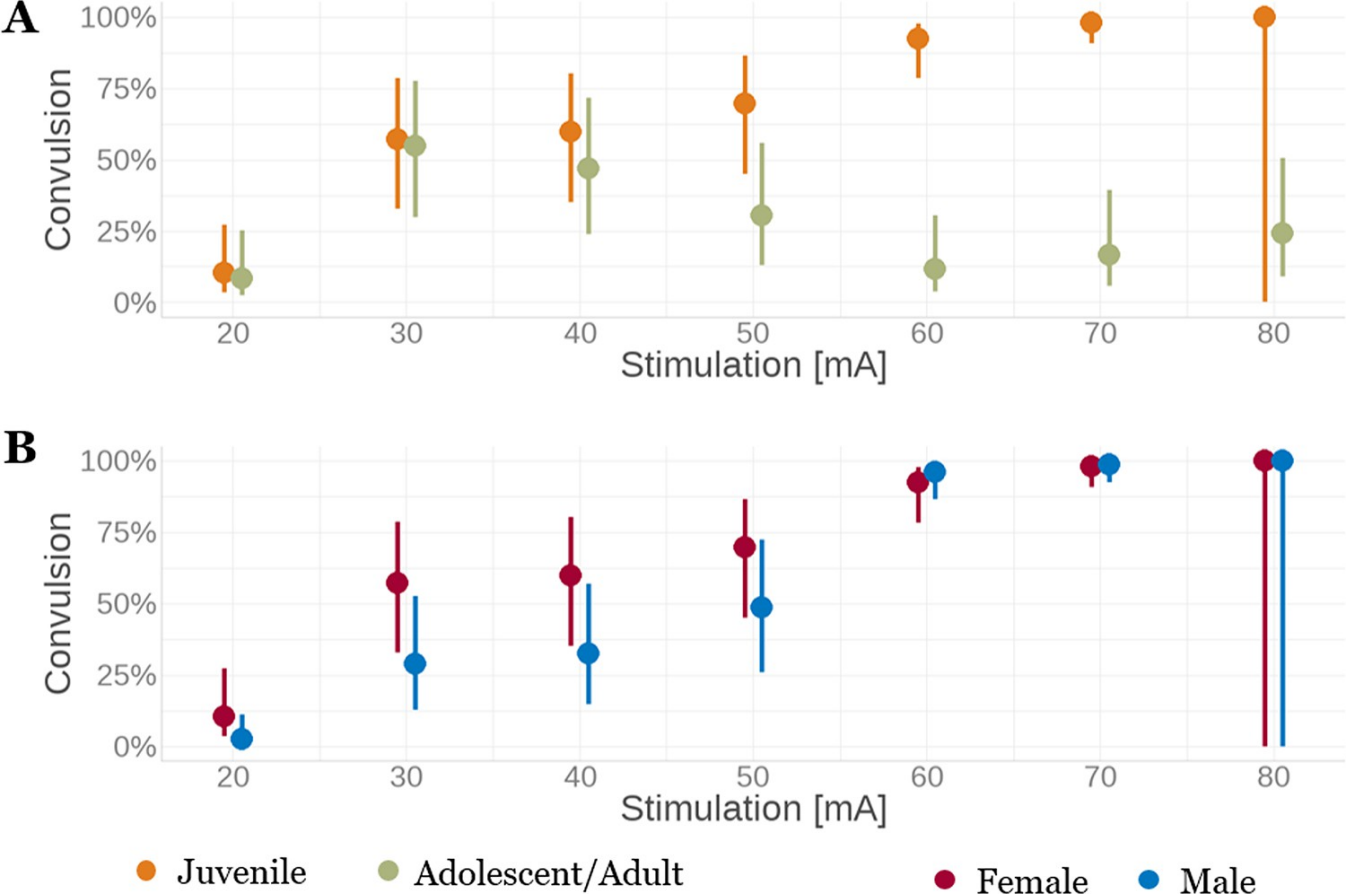
Predicted probabilities of the occurrence of motor seizures depending on stimulation intensity. Panel A depicts the age-related differences in the probability of development of convulsive seizures (Racine 3–5) between juvenile (P15-P25; orange) and adolescent/adult (P31-P60; green) rats at each stimulation intensity. Panel B shows the effects of sex (males in blue, females in red) on predicted probabilities of motor seizures. In both panels, the error bars represent 95% confidential interval with an estimated mean computed from the statistical model stated in Material and Methods.

To reflect this developmental pattern of convulsive seizures occurrence, animals were divided into two developmental categories: juvenile (P15-P25) and adolescent/adult (P31-P60) for assessment of the possible association of seizure probability with sex and developmental category. A logistic mixed-effects regression was employed for the assessment of the risk of motor seizure (score 3–5) development at individual stimulation intensities in these two developmental categories (Fig 3A). Type II ANOVA assessed the association and the results indicated the significant association of the convulsion occurrence with the level of maturation, the stimulation intensity and the interaction of stimulation with the level of maturation or sex (p < 0.01 for all). The significant interactions indicate that the effects of age or sex depend on the intensity of the stimulation. Specifically, our data show that starting at 50mA the probability of convulsion occurrence is higher in juveniles compared to adolescent/adult rats and the difference in risk of convulsion development increases with stimulation intensity. In contrast to the developmental categories, sex did not affect the probability of motor seizure development (Fig 3B). The comparisons between the two developmental categories discovered significant differences between juvenile and adolescent/adult animals regardless of sex at 50 mA and higher stimulation (p<0.001 for all). The outcomes implied that the probability of convulsions increased in juveniles from 20 mA to 80 mA until nearly all animals had convulsions, while, in adolescence/adult, the probability of convulsions started to decrease at 40 mA and decreased to ~25% at 80 mA (Fig 3A). In contrast, the comparison between the sexes did not reveal any significant difference at any stimulation point.

### 3.4. Duration of convulsive seizures

The three younger groups exhibited similar pattern as the incidence of seizures, i.e.an increase in duration of seizures with increasing intensity of stimulation (S1 Fig). In older groups this relationship was observed only in female 25-day-old rats. Again, adolescent/adult rats (P35-P60) had an erroneous relation of duration of seizures to intensity of stimulation. Seizures elicited by higher stimulation intensities (>50 mA) tended to be shorter in P18 and P21 females, but the lower number of animals precluded statistical analysis for this relationship.

A possible effect of interstimulation interval on irregular responses in older animals was excluded by pilot experiments with intervals up to one hour in 25-day-old rats. The results demonstrated the same irregular appearance of convulsive seizures.

### 3.5. Effects of age and sex on response to stimulation

**Fig 4:**
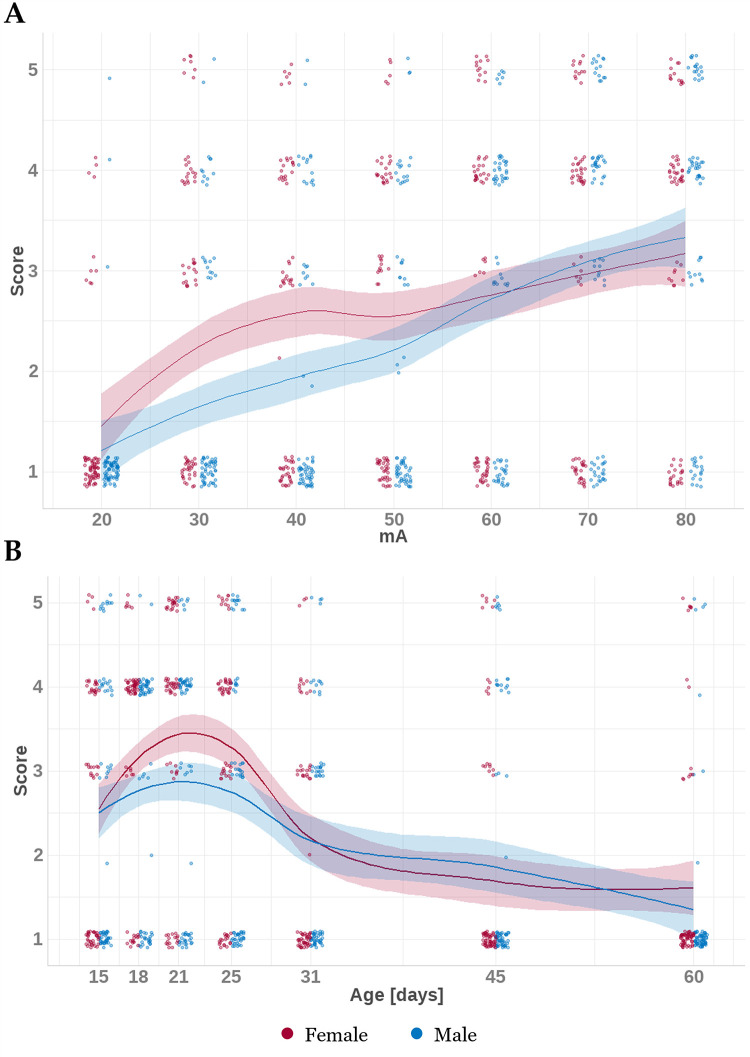
Visualization of association between score and threshold intensity and/or age. The multiplot visualizes the association of score (1–5) and (A) stimulation threshold or (B) age in males (blue) or females (red). In both panels, each data point represents a measured score at the respective threshold or age, while the line with a shaded ribbon signifies the trend along with 95% confidence intervals. The plots indicate that the score positively correlates with the threshold in both sexes; however, the score increases only in females up to the 25^th^ day, after which it decreases in both sexes until the 60^th^ day.

Juvenile animals (P15 –P25) of both sexes were more prone to convulsive seizures after stimulation with the higher current (50mA and more), whereas in adolescent/adult animals (P31-P60) convulsions appeared irregularly regardless of stimulation intensities. Sex did not affect the risk of convulsive seizure development even though threshold stimulation intensities necessary for convulsive seizure induction tended to be lower in females of all age groups and score values tended to be higher in juvenile females than in males. Also seizure severity expressed as a score appear to be higher after lower intensity stimulation (30-50mA) in juvenile females (Fig 4).

These data suggest that the estrous cycle in 31-day-old and older female rats was likely not consequential because the variability of seizure scores was the same as in prepubertal rats.

## 4. Discussion

The youngest group in our study was 15 days old because younger animals do not yet have eyelids open. To perform testing in younger animals requires surgical separation of eyelids causing additional stress for animals. This additional variable, or the alternative of stimulation through closed eyelids, precludes appropriate comparisons with the data presented herein.

Ontogenetic development exhibited an outlined U (or rather inverted J) shape of threshold intensities. This curve is similar to PTZ-induced generalized seizures where the lowest doses of this convulsant drug elicited generalized tonic-clonic seizures in the third postnatal week in both male and female rats [31]. In spite of identical semiology with 6-Hz convulsions [31], minimal clonic seizures elicited by pentylenetetrazol in rats 18 and more days old (minimal Metrazol seizures in the older literature) exhibit different age profile with decreasing threshold convulsant doses from the age of 18 days till adulthood. Identical semiology with minimal clonic seizures generated in the basal forebrain [32] might indicate the site of origin of 6-Hz induced seizures in the same strucutres. As concerns involvement of limbic structures, participation of amygdala was demonstrated in adult mice since the first seizures whereas activation of hippocampus was visible only after the fourth suprathreshold stimulation [33]. Our experiments started with subthreshold stimulation intensities and stimulation was repeated in 20-min intervals (in contrast to 72 hours in Giordano`s experiments) therefore involvement of hippocampus might be taken into account only for highest stimulation intensities but „stunned”position described as typical for hippocampal activity was not present in our animals.

Our developmental data in the 6 Hz model are also in contradiction with the development of sensitivity to systemically administered kainic acid. Minimal clonic seizures were present after behavioral automatisms and the dose of kainic aid necessary for elicitation of seizures decreased with age [19]. The effects of intracortical and intrahippocampal administration of kainic acid were studied in rat pups using EEG [20]. This study demonstrated the presence of epileptic activity even in the youngest group (4–7 days old rat pups) and the start of this activity at the site of injection. Epileptic afterdischarges elicited by hippocampal stimulation exhibited decreasing threshold intensities of stimulation from postnatal day 12 till day 18, then the value was the same in 45- and 60-day-old rats [34]. This comparison of different models of temporal lobe seizures in developing rats demonstrates high variability according to the epileptogenic agents.

We presumed that the constant presence of seizures in 21-day-old and younger rats is due to immature postictal depression like in cortically-induced seizures [35] and that irregular appearance of seizures in older rats might among other factors express the presence and persistence of postictal refractoriness, i.e. maturation of mechanisms arresting seizures. Pilot experiments with longer interstimulation intervals (up to one hour) in 25-day-old rats demonstrating irregular appearance of convulsive seizures did not support the decisive role of postictal depression. Therefore, it is necessary to analyze this phenomenon in the future. On the other hand, repeated stimulations in adult mice might result in regular kindling phenomenon [8,9] but intervals between stimulation series were in the order of hours (e.g. twice daily [11]). Seizure duration and severity showed a clear stimulus intensity—response relationship through 18–21 days (Fig 2). The missing relationship between stimulation intensity and severity of seizures in animals 31 and more days old was seen in both sexes.

Differences between male and female rat pups suggest higher excitability of female rats. The lower threshold for elicitation of seizures in 25-day-old female rats might not be due to the estrous cycle which started in our breeding of Wistar rats after postnatal day 30. This is in agreement with the literature presenting postnatal day 30 as the earliest date [36]. Therefore, stage of the estrous cycle must be taken into account in female rats more than 30 days old. It was not assessed in the present study, but a similar variability of results of female and male rats speaks against the high significance of the estrous stage. The differences between the two sexes demonstrated in some epileptic phenomena in various age groups shall be taken as a reason against performing experiments in nests composed of pups of both sexes.

## 5. Conclusions

Our results indicate the reliability of the 6-Hz model for studies of neurotransmitter mechanisms (like Jahan et al. [37]) and testing of anticonvulsant drugs in rat pups younger than three weeks like in adult animals [38]. The irregular results from older animals represent a serious limitation and they need further analysis. We would like to start with various intervals between stimulation series.

## Supporting information

S1 Fig Data for seven age groups of male (blue) and female (red) rats. Left column from top to bottom: Rats aged 15, 18, 21, and 25 postnatal days; right column: Animals 31, 45, and 60 days old. Median and 95% confidence limits are presented. Each graph: x-axis: Intensity of stimulation current in mA; y-axis: Duration of seizures in seconds.
(DOCX)

S1 Table The table presents a result of ANOVA analysis conducted to evaluate the significant influence of sex, age and their interaction on the threshold stimulation intensity necessary to induce motor seizures (score 3–5). The findings underscore the significant roles played by sex and age in determining the threshold stimulation, with no notable interaction effect between them.
(DOCX)

S2 Table This table illustrates sex-related differences in the threshold stimulation intensities within distinct age groups. The estimated differences are represented on a logarithmic scale. The results highlight statistically significant disparities between sexes at ages P18 and P60.
(DOCX)

S3 Table Analysis of factors influencing convulsions. The table shows the results of Type II ANOVA computed from a generalized linear-mixed effect models, assessing the impact of stage, gender, stimulation, and their interactions on the presence of convulsions. The results reveal a noteworthy association between the motor seizure score (3–5) and all variables, except sex, which did not show a significant association. Significant interactions between stimulation and both stage and gender were followed up with Tukey’s post-hoc analyses (Supplementary Tab 4).
(DOCX)

S4 Table Table of differences within developmental category (A) and sex (B) at each stimulation intensities. Tables show differences (A) between juvenile and adolescent/adult animals and (B) between males and females within distinct stimulation intensities. The estimated difference represents the difference between developmental category or sex at the log scale.
(DOCX)

S5 Table Threshold intensities of stimluation current. Left column–age in days; middle column–female rats; right column–male rats. Data for both female and male rats exhibit mean intensity and stadard deviation in mA in left part and number of animals in the right part.
(DOCX)

S6 Table Stimulation intensity/ number of animals with convulsions. Upper half–females, lower half–males. Age of the animals is presented in the left column, columns 3–9 show individual intensities of current from 20 to 80 mA.
(DOCX)

S7 Table Seizure severity (score) in individual age and stimulation intensity groups. Score is expressed as mean SD. Details as in S6 Table.
(DOCX)
