# Supplementary material for: Seizures elicited by transcorneal 6 Hz stimulation in developing rats
Source: PLoS One. 2025 Jan 3;20(1):e0313681. doi: 10.1371/journal.pone.0313681 (PMC11698314; doi:10.1371/journal.pone.0313681)
Supplement: S1 Fig — Left column from top to bottom: Rats aged 15, 18, 21, and 25 postnatal days; right column: Animals 31, 45, and 60 days old. Median and 95% confidence limits are presented. Each graph: x-axis: Intensity of stimulation current in mA; y-axis: Duration of seizures in seconds. (DOCX) [file pone.0313681.s001.docx]

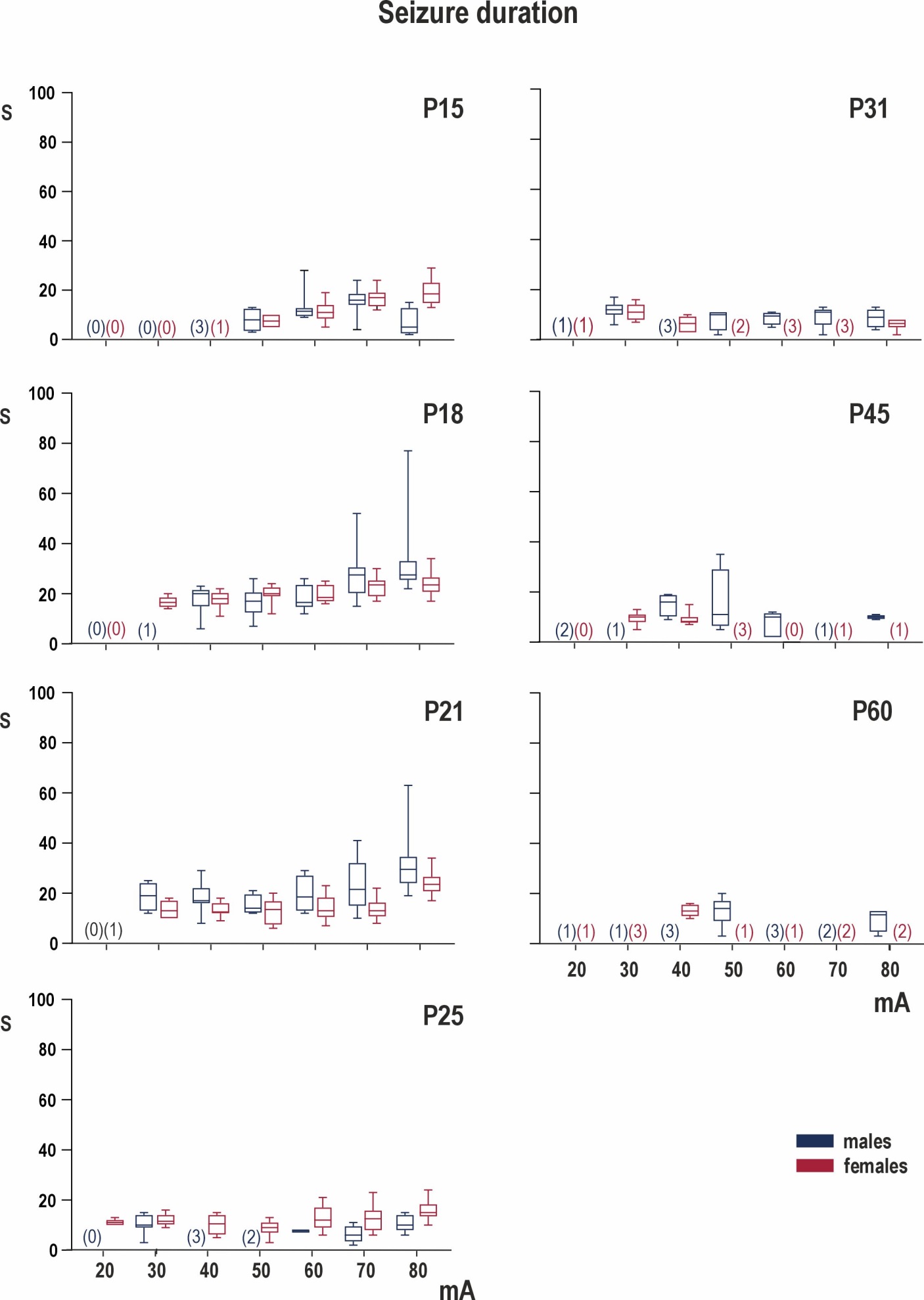


**Suipplementary Figure 1.** Data for seven age groups of male (blue) and female (red) rats. Left column from top to bottom: rats aged 15, 18, 21, and 25 postnatal days; right column: animals 31, 45, and 60 days old. Median and 95% confidence limits are presented. Each graph: x-axis: intensity of stimulation current in mA; y-axis: duration of seizures in seconds.
