# Supplementary material for: Seizures elicited by transcorneal 6 Hz stimulation in developing rats
Source: PLoS One. 2025 Jan 3;20(1):e0313681. doi: 10.1371/journal.pone.0313681 (PMC11698314; doi:10.1371/journal.pone.0313681)
Supplement: S1 Table — The findings underscore the significant roles played by sex and age in determining the threshold stimulation, with no notable interaction effect between them. (DOCX) [file pone.0313681.s002.docx]

**Supplementary Table 1** – The table presents a result of ANOVA analysis conducted to evaluate the significant influence of sex, age and their interaction on the threshold stimulation intensity necessary to induce motor seizures (score 3-5). The findings underscore the significant roles played by sex and age in determining the threshold stimulation, with no notable interaction effect between them.

|  | **Chi-square** | **df** | **p-value** |
| --- | --- | --- | --- |
| Age | 176.05 | 6 | <0.001 |
| Sex | 13.56 | 1 | <0.001 |
| Age:Sex | 11.43 | 6 | 0.076 |
