# Supplementary material for: Seizures elicited by transcorneal 6 Hz stimulation in developing rats
Source: PLoS One. 2025 Jan 3;20(1):e0313681. doi: 10.1371/journal.pone.0313681 (PMC11698314; doi:10.1371/journal.pone.0313681)
Supplement: S2 Table — The estimated differences are represented on a logarithmic scale. The results highlight statistically significant disparities between sexes at ages P18 and P60. (DOCX) [file pone.0313681.s003.docx]

**Supplementary Table 2** – This table illustrates sex-related differences in the threshold stimulation intensities within distinct age groups. The estimated differences are represented on a logarithmic scale. The results highlight statistically significant disparities between sexes at ages P18 and P60.

| **Contrasts** | **Estimated difference** | **5% CI** | **95% CI** | **Z-ratio** | **p-value** |
| --- | --- | --- | --- | --- | --- |
| 15 days - Male-Female | 2.00 | -4.953 | 8.953 | 0.569 | 0.570 |
| 18 days - Male-Female | 10.00 | 3.047 | 16.953 | 2.846 | 0.005 |
| 21 days - Male-Female | 3.00 | -3.953 | 9.953 | 0.854 | 0.395 |
| 25 days - Male-Female | 8.00 | 1.047 | 14.953 | 2.277 | 0.024 |
| 31 days - Male-Female | 2.11 | -5.032 | 9.255 | 0.585 | 0.560 |
| 45 days - Male-Female | -2.00 | -8.953 | 4.953 | -0.569 | 0.570 |
| 60 days - Male-Female | 11.10 | 4.147 | 18.053 | 3.16 | 0.002 |
