# Supplementary material for: Seizures elicited by transcorneal 6 Hz stimulation in developing rats
Source: PLoS One. 2025 Jan 3;20(1):e0313681. doi: 10.1371/journal.pone.0313681 (PMC11698314; doi:10.1371/journal.pone.0313681)
Supplement: S3 Table — The table shows the results of Type II ANOVA computed from a generalized linear-mixed effect models, assessing the impact of stage, gender, stimulation, and their interactions on the presence of convulsions. The results reveal a noteworthy association between the motor seizure score (3–5) and all variables, except sex, which did not show a significant association. Significant interactions between stimulation and both stage and gender were followed up with Tukey’s post-hoc analyses (Supplementary Tab 4). (DOCX) [file pone.0313681.s004.docx]

**Supplementary Tab 3.** **Analysis of Factors Influencing Convulsions -** The table shows the results of Type II ANOVA computed from a generalized linear-mixed effect models, assessing the impact of stage, gender, stimulation, and their interactions on the presence of convulsions. The results reveal a noteworthy association between the motor seizure score (3-5) and all variables, except sex, which did not show a significant association. Significant interactions between stimulation and both stage and gender were followed up with Tukey's post-hoc analyses (Supplementary Tab 4).

|  | **Chi-square** | **df** | **p-value** |
| --- | --- | --- | --- |
| Stimulation intensity | 59.09 | 6 | <0.001 |
| Sex | 0.94 | 1 | 0.331 |
| Developmental category | 45.74 | 1 | <0.001 |
| Stimulation intensity x developmental category | 74.23 | 6 | <0.001 |
| Stimulation intensity x sex | 17.30 | 6 | 0.008 |
