# Supplementary material for: Seizures elicited by transcorneal 6 Hz stimulation in developing rats
Source: PLoS One. 2025 Jan 3;20(1):e0313681. doi: 10.1371/journal.pone.0313681 (PMC11698314; doi:10.1371/journal.pone.0313681)
Supplement: S4 Table — Table of differences within developmental category (A) and sex (B) at each stimulation intensities. Tables show differences (A) between juvenile and adolescent/adult animals and (B) between males and females within distinct stimulation intensities. The estimated difference represents the difference between developmental category or sex at the log scale. (DOCX) [file pone.0313681.s005.docx]

**Supplementary Tab 4.** **Table of differences within developmental category (A) and sex (B) at each stimulation intensities** - Tables show differences (A) between juvenile and adolescent/adult animals and (B) between males and females within distinct stimulation intensities. The estimated difference represents the difference between developmental category or sex at the log scale.

| **A** | **Differences** | **log(OR)** | **5% CI** | **95% CI** | **Z-ratio** | **p-value** |
| --- | --- | --- | --- | --- | --- | --- |
|  | 20 mA - Adolescence/Adult- Juvenile | -0.229 | -1.49 | 1.031 | -0.357 | 0.721 |
|  | 30 mA - Adolescence/Adult- Juvenile | -0.094 | -0.882 | 0.694 | -0.234 | 0.815 |
|  | 40 mA - Adolescence/Adult- Juvenile | -0.517 | -1.309 | 0.275 | -1.279 | 0.201 |
|  | 50 mA - Adolescence/Adult- Juvenile | -1.653 | -2.479 | -0.827 | -3.922 | <0.001 |
|  | 60 mA - Adolescence/Adult- Juvenile | -4.51 | -5.62 | -3.399 | -7.959 | <0.001 |
|  | 70 mA - Adolescence/Adult- Juvenile | -5.424 | -6.87 | -3.979 | -7.354 | <0.001 |
|  | 80 mA - Adolescence/Adult- Juvenile | -19.916 | -2113.911 | 2074.079 | -0.019 | 0.985 |
|  |  |  |  |  |  |  |
|  |  | | | | | |
| **B** | **Differences** | **log(OR)** | **5% CI** | **95% CI** | **Z-ratio** | **p-value** |
|  | 20 mA - Male-Female | -1.438 | -3.239 | 0.363 | -1.565 | 0.118 |
|  | 30 mA - Male-Female | -1.196 | -2.545 | 0.152 | -1.738 | 0.082 |
|  | 40 mA - Male-Female | -1.128 | -2.478 | 0.221 | -1.639 | 0.101 |
|  | 50 mA - Male-Female | -0.877 | -2.237 | 0.482 | -1.265 | 0.206 |
|  | 60 mA - Male-Female | 0.667 | -0.852 | 2.186 | 0.861 | 0.389 |
|  | 70 mA - Male-Female | 0.327 | -1.259 | 1.913 | 0.404 | 0.686 |
|  | 80 mA - Male-Female | 0.6 | -1.024 | 2.224 | 0.724 | 0.469 |
