# Supplementary material for: Seizures elicited by transcorneal 6 Hz stimulation in developing rats
Source: PLoS One. 2025 Jan 3;20(1):e0313681. doi: 10.1371/journal.pone.0313681 (PMC11698314; doi:10.1371/journal.pone.0313681)
Supplement: S5 Table — Left column–age in days; middle column–female rats; right column–male rats. Data for both female and male rats exhibit mean intensity and stadard deviation in mA in left part and number of animals in the right part. (DOCX) [file pone.0313681.s006.docx]

Supplementary table 5: Thresholds

| **Age group** | **Females** | | **Males** | |
| --- | --- | --- | --- | --- |
|  | mean±SD | Number of animals in group | mean±SD | Number of animals in group |
| **P15** | 54.0±8.4 | 10 | 56.0±8.4 | 10 |
| **P18** | 34.0±5.2 | 10 | 44.0±8.4 | 10 |
| **P21** | 32.0±6.3 | 10 | 35.0±5.3 | 10 |
| **P25** | 23.0±4.8 | 10 | 31.0±3.2 | 10 |
| **P31** | 28.9±3.3 | 9 | 31.0±5.7 | 10 |
| **P45** | 33.0±4.8 | 10 | 31.0±13.7 | 10 |
| **P60** | 36.9±8.2 | 10 | 48.0±14.0 | 10 |
