# Supplementary material for: Seizures elicited by transcorneal 6 Hz stimulation in developing rats
Source: PLoS One. 2025 Jan 3;20(1):e0313681. doi: 10.1371/journal.pone.0313681 (PMC11698314; doi:10.1371/journal.pone.0313681)
Supplement: S6 Table — Upper half–females, lower half–males. Age of the animals is presented in the left column, columns 3–9 show individual intensities of current from 20 to 80 mA. (DOCX) [file pone.0313681.s007.docx]

**Supplementary table 6**: Seizure severity (score). Mean ± standard deviation in individual age – sex –stimulation intensity groups

| **Age group** | **Sex** | **Stimulation intensity** | | | | | | |
| --- | --- | --- | --- | --- | --- | --- | --- | --- |
|  |  | **20mA** | **30mA** | **40mA** | **50mA** | **60mA** | **70mA** | **80mA** |
| **P15** | **Female** | 1.0±0.0 | 1.0±0.0 | 1.2±0.6 | 2.3±1.2 | 3.6±1.2 | 4.1±0.3 | 3.8±0.6 |
| **P18** |  | 1.0±0.0 | 2.5±1.3 | 3.7±0.5 | 3.9±1.1 | 4.2±0.4 | 4.1±0.3 | 4.1±0.3 |
| **P21** |  | 1.2±0.6 | 2.8±1.3 | 3.3±1.3 | 3.3±1.3 | 4.5±0.5 | 4.6±0.5 | 4.7±0.5 |
| **P25** |  | 2.7±1.2 | 3.1±1.2 | 2.2±1.6 | 2.8±1.3 | 3.3±1.7 | 3.3±1.4 | 4.4±0.5 |
| **P31** |  | 1.3±0.9 | 3.4±1.2 | 2.4±1.3 | 1.5±1.1 | 1.8±1.3 | 1.7±1.2 | 2.4±1.3 |
| **P45** |  | 1.0±0.0 | 3.2±1.7 | 2.9±1.5 | 1.9±1.5 | 1.0±0.0 | 1.2±0.6 | 1.3±0.9 |
| **P60** |  | 1.2±0.6 | 1.8±1.7 | 1.9±1.5 | 1.7±1.5 | 1.2±0.6 | 2.0±1.7 | 1.4±0.6 |
| **P15** | **Male** | 1.0±0.0 | 1.0±0.0 | 1.2±0.6 | 1.3±0.7 | 3.9±0.7 | 4.5±0.5 | 4.0±0.8 |
| **P18** |  | 1.0±0.0 | 1.2±0.6 | 2.4±1.3 | 3.5±1.0 | 4.0±0.0 | 4.1±0.3 | 4.1±0.3 |
| **P21** |  | 1.0±0.0 | 2.2±1.3 | 2.7±1.3 | 2.1±1.4 | 4.1±0.3 | 4.2±0.6 | 4.2±0.4 |
| **P25** |  | 1.0±0.0 | 3.3±0.9 | 1.6±1.0 | 1.4±0.8 | 2.4±1.3 | 3.7±1.3 | 4.9±0.3 |
| **P31** |  | 1.2±0.6 | 6.0±1.6 | 2.0±1.6 | 2.0±1.3 | 1.8±1.0 | 2.1±1.2 | 2.8±1.4 |
| **P45** |  | 1.7±1.5 | 1.3±0.9 | 2.3±1.7 | 2.1±1.6 | 2.8±1.9 | 1.3±0.9 | 1.9±1.2 |
| **P60** |  | 1.0±0.0 | 1.0±0.0 | 1.3±0.9 | 2.1±1.7 | 1.0±0.0 | 1.6±1.3 | 1.4±1.3 |
