# Supplementary material for: Seizures elicited by transcorneal 6 Hz stimulation in developing rats
Source: PLoS One. 2025 Jan 3;20(1):e0313681. doi: 10.1371/journal.pone.0313681 (PMC11698314; doi:10.1371/journal.pone.0313681)
Supplement: S7 Table — Score is expressed as mean SD. Details as in S6 Table. (DOCX) [file pone.0313681.s008.docx]

Supplementary table 7

| **Age group** | **Sex** | **Stimulation intensity/ number of animals with convulsions** | | | | | | |
| --- | --- | --- | --- | --- | --- | --- | --- | --- |
|  |  | **20mA** | **30mA** | **40mA** | **50mA** | **60mA** | **70mA** | **80mA** |
| **P15** | F | *n=0* | *n=0* | *n=2* | *n=2* | 8.9±2.1  *n=10* | 16.1±2.5  *n=10* | 19.4±2.9  *n=10* |
| **P18** |  | *n=0* | 16.7±2.3  *n=6* | 17.8±3.3  *n=10* | 19.9±3.4  *n=9* | 19.7±3.4  *n=10* | 22.6±3.9  *n=10* | 23.9±4.7  *n=10* |
| **P21** |  | *n=1* | 13.3±3.1  *n=7* | 13.3±2.9  *n=7* | 12.8±5.0  *n=6* | 14.1±4.9  *n=10* | 13.7±3.9  *n=10* | 14.3±3.4  *n=10* |
| **P25** |  | 11.3±1.1  *n=7* | 12.1±2.4  *n=8* | 10.3±4.1  *n=4* | 8.6±3.5  *n=7* | 12.9±5.3  *n=7* | 12.6±5.5  *n=8* | 15.6±4.5  *n=10* |
| **P31** |  | *n=1* | 11.0±3.3  *n=9* | 6.7±2.1  *n=6* | *n=2* | *n=3* | *n=2* | 6.2±2.2  *n=6* |
| **P45** |  | *n=0* | 9.3±2.6  *n=7* | 9.4±2.7  *n=7* | 8.7±3.8  *n=4* | *n=0* | *n=1* | *n=1* |
| **P60** |  | *n=1* | *n=3* | 13.2±2.4  *n=5* | *n=1* | *n=1* | *n=1* | *n=1* |
| **P15** | **M** | *n=0* | *n=0* | *n=2* | *n=2* | 8.9±2.1  n=10 | 16.1±2.5  n=10 | 19.4±2.9  n=10 |
| **P18** |  | *n=0* | *n=1* | 18.0±6.1  n=6 | 16.8±5.7  n=9 | 18.3±4.8  n=10 | 27.5±10.3  n=10 | 33.0±16.0  n=10 |
| **P21** |  | *n=0* | 18.6±5.6  n=5 | 18.1±6.4  n=7 | 15.3±4.0  n=4 | 19.9±6.4  n=10 | 23.1±10.2  n=10 | 31.8±12.1  n=10 |
| **P25** |  | *n=0* | 10.6±3.6  n=9 | n=3 | n=2 | 7.7±0.5  n=6 | 6.6±3.2  n=9 | 10.7±3.1  n=10 |
| **P31** |  | *n=0* | 11.6±3.5  n=7 | n=3 | 8.3±4.3  n=4 | 8.8±2.3  n=5 | 9.4±4.3  n=5 | 9.1±3.2  n=7 |
| **P45** |  | *n=2* | *n=1* | 15±4.5  *n=4* | 13±8.5  *n=4* | 7.4±5.0  *n=5* | *n=1* | 10±0.8  *n=4* |
| **P60** |  | *n=1* | *n=0* | *n=3* | 10.8±6.1  *n=4* | *n=2* | *n=2* | *n=2* |
